# *Bartonella* gene transfer agent: Evolution, function, and proposed role in host adaptation

**DOI:** 10.1111/cmi.13068

**Published:** 2019-07-09

**Authors:** Maxime Québatte, Christoph Dehio

**Affiliations:** ^1^ Biozentrum University of Basel Basel Switzerland

**Keywords:** *Bartonella*, gene transfer agent (GTA), horizontal gene transfer (HGT), host adaptation, Muller's ratchet, type IV secretion systems (T4SS)

## Abstract

The processes underlying host adaptation by bacterial pathogens remain a fundamental question with relevant clinical, ecological, and evolutionary implications. Zoonotic pathogens of the genus *Bartonella* constitute an exceptional model to study these aspects. *Bartonellae* have undergone a spectacular diversification into multiple species resulting from adaptive radiation. Specific adaptations of a complex facultative intracellular lifestyle have enabled the colonisation of distinct mammalian reservoir hosts. This remarkable host adaptability has a multifactorial basis and is thought to be driven by horizontal gene transfer (HGT) and recombination among a limited genus‐specific pan genome. Recent functional and evolutionary studies revealed that the conserved *Bartonella* gene transfer agent (BaGTA) mediates highly efficient HGT and could thus drive this evolution. Here, we review the recent progress made towards understanding BaGTA evolution, function, and its role in the evolution and pathogenesis of *Bartonella* spp. We notably discuss how BaGTA could have contributed to genome diversification through recombination of beneficial traits that underlie host adaptability. We further address how BaGTA may counter the accumulation of deleterious mutations in clonal populations (Muller's ratchet), which are expected to occur through the recurrent transmission bottlenecks during the complex infection cycle of these pathogens in their mammalian reservoir hosts and arthropod vectors.

## 1 INTRODUCTION

*Bartonella* species are arthropod‐borne, facultative intracellular pathogens that are highly adapted to their specific mammalian reservoir host(s) based on a multitude of intimate interactions at the pathogen–host interface (Harms & Dehio, 2012; Siamer & Dehio, 2015; Wagner & Dehio, 2019). This host specificity and the pathogen's capacity for genetic diversification has driven major adaptive radiations in at least two *Bartonella* lineages. Notably, these radiations occurred after the acquisition of type IV secretion systems (T4SSs) and, possibly, further pathogenesis factors (e.g., autotransporter‐type adhesins) that mediate crucial host‐specific interactions (Engel et al., 2011; Harms et al., 2017). Together with homologous recombination, horizontal gene transfer (HGT) facilitates the combination of beneficial traits evolved in different isolates within one genome (Hall, Brockhurst, & Harrison, 2017). It should thus be a crucial factor in the ongoing process of host adaptation of the multifactorial pathogenesis strategy of the *Bartonellae*. On the other hand, the infection cycle of *Bartonella* contains major transmission bottlenecks, both at the level of the arthropod vector and while breaching their mammalian host's barriers (Harms & Dehio, 2012; Siamer & Dehio, 2015). From this perspective, HGT and homologous recombination in clonal populations may contribute to maintaining genome integrity and counter Muller's ratchet, that is, the accumulation of deleterious mutations in isolated bacterial populations (Muller, 1964; Takeuchi, Kaneko, & Koonin, 2014). In this context, we demonstrated that a *Bartonella*‐specific gene transfer agent (BaGTA) mediates high‐frequency genome‐wide recombination (Québatte et al., 2017). In this microreview, we will discuss the recent progress made towards understanding the role of BaGTA, with a special focus on its function and putative contribution(s) to host adaptation and to the radiating evolution of these fascinating pathogenic bacteria. We will address the relative weight of the different HGT mechanisms present in the *Bartonellae* and further highlight the unique properties of BaGTA in contrast to the archetypal system of *Rhodobacter capsulatus* (RcGTA).

## 2 ORIGIN AND EVOLUTION OF BaGTA

Gene transfer agents (GTAs) are commonly defined as virus‐like particles that mediate DNA transfer from the organism that encodes them into competent recipient cells (recently reviewed in Grull, Mulligan, & Lang, 2018; Lang, Westbye, & Beatty, 2017). As such, they constitute an important mechanism underlying HGT in addition to conjugation, natural competence, and bacteriophage‐mediated transduction (Sun, 2018; von Wintersdorff et al., 2016). It is generally accepted that GTAs originate from defective temperate prophages, in a process that has been conceptualised as “prophage domestication” (Bobay, Touchon, & Rocha, 2014; Harrison & Brockhurst, 2017; Olszak, Latka, Roszniowski, Valvano, & Drulis‐Kawa, 2017). Their independent emergence in different bacterial lineages constitutes a striking example of convergent evolution (Lang et al., 2017), which can be compared with the recurrent evolution of host‐interacting secretion systems from conjugative systems or from bacterial flagellum (Abby & Rocha, 2012; Frank, Alsmark, Thollesson, & Andersson, 2005).

**Figure 1:**
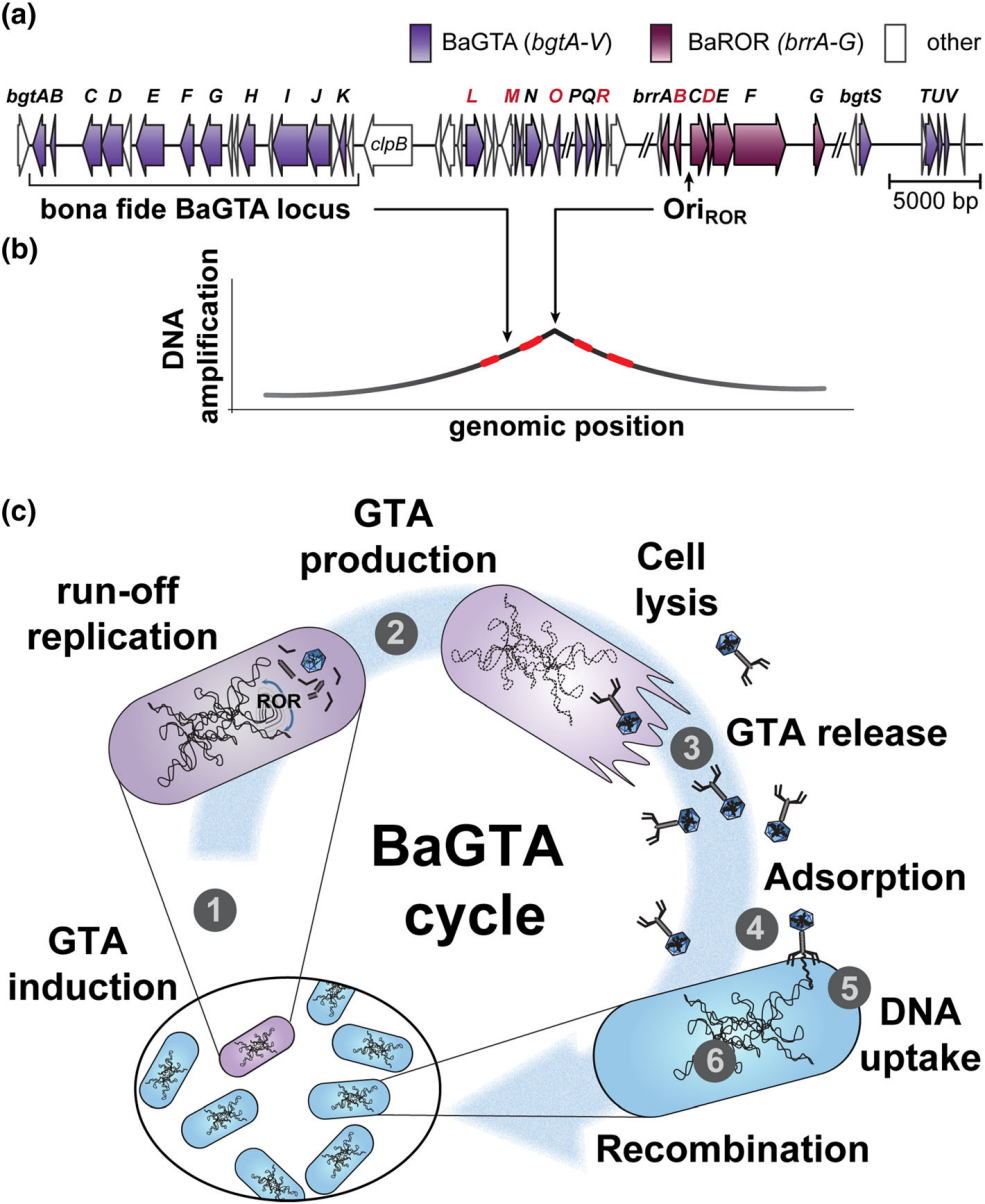
BaGTA, BaROR, and DNA transfer cycle. Model of the BaGTA cycle based on the data published in Québatte et al. (2017). (a) Chromosomal organisation (*Bartonella henselae*). The BaGTA genes (*bgtA‐V*) and the ROR‐associated genes (*brrA‐G*) are organised within an 80‐kb‐long genome segment, with a cluster of 11 genes (*bgtA‐K*) representing the bona fide BaGTA locus. The run‐off replication origin (Ori_ROR_) is indicated. Genes that are essential for *Bartonella* growth (*bgtL*, *M*, *O*, *R* and *brrB*, *D*) are indicated in red. (b) ROR activity. BaROR induction is initiated at Ori_ROR_ and results in a bidirectional amplification of adjacent DNA. Highlighted in red are regions encoding host adaptation factors, including T4SSs and T5SSs. (c) BaGTA cycle. Donor activities: (1) activation: stochastic BaGTA activation in a subpopulation of growing *Bartonella*. BaGTA induction is restricted to fast‐growing bacteria via a regulatory wiring to cellular ppGpp levels. Cumulative induction can reach up to 15% BaGTA induction in *B. henselae* using in‐vitro conditions mimicking host interaction. (2) Run‐off replication and BaGTA induction: BaGTA expression is plausibly coupled to ROR activation (see main text). Assembly of the BaGTA particles included cleavage of the amplified DNA into 14‐kb fragments and subsequent packaging by a yet unknown mechanism. (3) Cell lysis and GTA release: Lysis occurs about 6 hr after the onset of BaGTA induction, possibly through lysozyme‐related proteins and results in the release of BaGTA particles. Recipient activities: (4) BaGTA adsorption: Particles dock to the recipient cells, albeit through a yet unresolved process. The involvement of the Tol/Pal complex restricts BaGTA uptake to a growing recipient. (5) DNA uptake: Incoming DNA is imported into the recipient's cytoplasm via the competence‐related components ComEC, ComM, ComF, and DprA, after the probable periplasmic delivery of the 14‐kb BaGTA DNA. (6) Homologous recombination: BaGTA DNA is incorporated into the recipient's chromosome via DprA and the host's homologous recombination machinery. The delay for phenotypic expression (monitored by antibiotic resistance conferred by the transfer of a resistance cassette) lagged about 160 min after peak induction of BaGTA in the donor cells

Thus far, two distinct GTAs have been identified in the α‐proteobacteria, namely, RcGTA and BaGTA. RcGTA is distributed in a broad range of species (Lang & Beatty, 2007; Shakya, Soucy, & Zhaxybayeva, 2017), whereas the distribution of BaGTA (Figure 1) is restricted to bacteria of the genus *Bartonella* (Berglund et al., 2009; Tamarit, Neuvonen, Engel, Guy, & Andersson, 2018). To date, further GTA paralogues have been identified in the sulfate reducer *Desulfovibrio desulfuricans* (Rapp & Wall, 1987), in the intestinal spirochaete *Brachyspira hyodysenteriae* (Humphrey, Stanton, Jensen, & Zuerner, 1997; Matson, Zuerner, & Stanton, 2007), and in the archaeal methanogen *Methanococcus voltae* (Bertani, 1999).

Named after *R. capsulatus* in which it was first identified (Marrs, 1974; Yen, Hu, & Marrs, 1979), RcGTA is the best studied GTA representative to date and is therefore often considered the archetypal system (for recent review, see Lang et al., 2017; Westbye, Beatty, & Lang, 2017). A recent analysis of RcGTA homologues supports its proposed phage origin and estimates that the chromosomal acquisition of this DNA transfer machinery within a subclade of the α‐proteobacteria occurred more than 500 million years ago (Shakya et al., 2017). RcGTAs harbour the clear signature of vertical transmission, although their evolution and spread has been further shaped by HGT (Hynes et al., 2016; Shakya et al., 2017). Interestingly, no homologues of RcGTA genes can be found in any representative of the genus *Bartonella*. This system was thus likely lost during the drastic reductions that shaped the genomes of these organisms (Batut, Andersson, & O'Callaghan, 2004; Segers, Kesnerova, Kosoy, & Engel, 2017). Instead, these bacteria have evolved an entirely distinct system, BaGTA, whose acquisition can be traced back to the last common ancestor of the *Bartonellaceae* (Tamarit et al., 2018). Thus, on the evolutionary scale, BaGTA appeared more recently than RcGTA. Not only does the acquisition of this system represent an ancestral feature of the genus *Bartonella*, but it also belongs to the narrow set of genes that is strictly conserved across all species of these arthropod‐borne zoonotic pathogens and their insect symbionts relatives (Guy et al., 2013; Tamarit et al., 2018). The chromosomal position of BaGTA was conserved, and sequence analysis supports that view that the core locus was primarily propagated by vertical transmission and consequently codiversified with *Bartonella* genomes (Tamarit et al., 2018). Interestingly, duplications and subsequent diversification of BaGTA genes have been observed in most species. In contrast to the BaGTA locus, these additional BaGTA‐like clusters have undergone independent losses as well as occasional HGT and recombination events (Tamarit et al., 2018), providing further insights into the dynamic genome architecture of the *Bartonellaceae* (see, for instance, Berglund et al., 2009; Engel & Dehio, 2009; Segers et al., 2017).

## 3 FUNCTION OF BaGTA

Although going back as early as the emergence of the *Bartonellaceae*, the function of BaGTA has undergone a drastic specialisation in the radiating lineage of the mammalian host‐associated species (Harms, Segers, et al., 2017; Segers et al., 2017). Indeed, from this point onwards, the BaGTA locus became linked to a phage‐derived origin of replication, known as the run‐off replication (ROR) gene cassette (Berglund et al., 2009; Figure 1). This acquisition has been strictly maintained in all modern *Bartonella* spp. (Guy et al., 2013; Tamarit et al., 2018) and coincides with the explosive adaptive radiation that characterizes this genus of hematotropic pathogens (Engel et al., 2011; Guy et al., 2013; Harms, Segers, et al., 2017). BaGTA and ROR were therefore proposed as the key innovations underlying the radiation process, a postulate bolstered by two important observations. First, there is a conserved association between these two loci and genes coding for toxins and secretion systems (Berglund et al., 2009; Guy et al., 2013), many of which have been shown to be essential for host interaction (Harms & Dehio, 2012; Wagner & Dehio, 2019). Second, the DNA encoding these host adaptation factors is overrepresented in BaGTA particles (Berglund et al., 2009) and consequently displays a higher transfer frequency into recipient cells compared with other regions of the chromosome (Québatte et al., 2017). More precisely, the frequency of DNA incorporation into BaGTA and subsequent transfer to a recipient is directly proportional to the distance to ROR. This further implies a functional coupling between ROR and BaGTA activities (Figure 1). Two experimental findings corroborate this model. First, disruption of the ROR replication origin (ORI_ROR_) or some of the conserved ROR genes abrogate BaGTA‐mediated DNA transfer (Québatte et al., 2017). Further, BaGTA‐mediated transfer is restricted to the ROR replicon, because episomal DNA is not transferred. Therefore, ROR acquisition by *Bartonella* not only resulted in a biased DNA selectivity by BaGTA but also evolved to become an integral component of the BaGTA machinery. The selectivity of BaGTA for its target DNA contrasts with the observation made for RcGTA, which was shown to uniformly package the *Rhodobacter* chromosome, with the exception of the RcGTA genes that were underrepresented (Hynes, Mercer, Watton, Buckley, & Lang, 2012). This raised the idea that RcGTA would function as a “generalist” system. However, that conclusion may not be generalisable to all RcGTAs, as complex, nonrandom pattern of DNA packaging was recently described for the homologue DsGTA in *Dinoroseobacter shibae* (Tomasch et al., 2018).

The nature of the transferred DNA undoubtedly hints at GTAs purpose(s) on the evolutionary timescale. On the other hand, a better understanding of GTAs function(s) on the timescale of the organism's life cycle may emerge by understanding the regulatory mechanisms involved in their control. Indeed, because GTA release is ultimately associated with the lysis of the donor cells (P. C. Fogg, Westbye, & Beatty, 2012; Hynes et al., 2012; Québatte et al., 2017), expression of these systems must be kept under stringent control, and their activation should be restricted to situation(s) where a beneficial outcome for the strain they are encoded in is maximised. Maybe not all too surprisingly, both BaGTA and RcGTA are directly controlled by their host's cell cycle. In *R. capsulatus*, RcGTA is preferentially activated in the stationary phase, during nutrient limitation and at high cell density. This specificity is achieved by a multilevel regulatory cascade that comprises (a) the master regulator of cell cycle CtrA; (b) the CckA–ChpT–DivL phosphorelay; (c) a quorum‐sensing module (all reviewed in Lang et al., 2017; Westbye, Beatty, et al., 2017); and (d) GafA, a recently characterized direct activator of RcGTA (P. C. M. Fogg, 2019). Physiologically, RcGTA expression is induced by starvation, via the secondary messenger ppGpp (Westbye, O'Neill, Schellenberg‐Beaver, & Beatty, 2017). A straightforward interpretation of this specific regulation scheme is that RcGTA is induced primarily at high cell density when resources have been exhausted, perhaps as an ultimate attempt to promote colonisation of new ecological niches. Importantly, the same regulatory circuit controls the expression of the genes required for uptake and assimilation of the transferred DNA, ultimately favouring kin selection. The strategy behind BaGTA expression appears almost opposite to the one described for RcGTA, possibly reflecting the fundamentally distinct lifestyles of the organisms in which they are encoded. Indeed, our work with *Bartonella henselae* indicates that particle production is restricted to healthy and growing cells and that this control is exerted via the sensing of intracellular ppGpp levels through a yet elusive mechanism (Québatte et al., 2017). Metabolic perturbations (mimicked in our experiments by transposon insertions into nutrient transporters) negatively affect BaGTA. They likely converge through ppGpp induction, and their effect supports optimal growth as a prerequisite condition. Regulation of GTA competence in recipient cells is also linked to cell division, notably through the involvement of the Tol–Pal *trans*‐envelope module (Québatte et al., 2017). Taken together, the growth requirements for both donors and recipients support the hypothesis that BaGTA promotes evolvability within the fittest bacterial subpopulation(s). These findings are perfectly in line with the proposed role for BaGTA in mediating the adaptive radiation process, although that may not be the full picture. For instance, this model does not explicitly account for the balance of the cost–benefit equation underlying the maintenance of these lytic elements. Because GTAs are not self‐transmissible, they cannot be considered selfish elements. Consequently, GTA maintenance in bacterial genomes must provide compensatory advantages to balance out the fitness cost their activation represents. Interestingly, a computational approach to the fitness cost generated by GTA expression concluded that the benefits of GTA‐mediated DNA transfer alone are not sufficient to explain maintenance (Redfield & Soucy, 2018). Although the model used in this study only considered a generalist mode of action (i.e., random DNA packaging), it still stresses the question of the selection pressure at work to prevent the emergence of cheaters within a bacterial population. In the case of BaGTA and *Bartonella*, the coupling of a ROR module to BaGTA may have been critical in tailoring the functionality of this system to the advantage of their pathogenic lifestyle. Moreover, the direct coupling of BaGTA induction to the fitness of the donor (Québatte et al., 2017) minimises the risk for the propagation of deleterious mutations. Nonetheless, an essential function for BaGTA during the *Bartonella* infection cycle would be a straightforward solution to ensure a strict conservation of this system in *Bartonella*. For instance, BaGTA‐induced antigenic variation by HGT between subclonal or closely related *Bartonella* isolates could be key to evade the selection pressure imposed by the host adaptive immunity. Although indications exist that such recombination events occur in vivo (see below), their impact on immune evasion has not been evaluated yet. Interestingly, previous work with *B. henselae* revealed that BaGTA is induced in the early stage of endothelial cell infection (Québatte et al., 2010). Moreover, transposon disruption of the BaGTA locus was shown to impair the induction of the VirB/D4 T4SS in *B. henselae* (Québatte, Dick, Kaever, Schmidt, & Dehio, 2013). Strikingly, the same effect was observed by disruption of competence genes involved in BaGTA uptake (e.g., DprA), indicating an intimate connection between BaGTA and regulation of *Bartonella* pathogenicity. Whether this cross‐regulation is conserved between all *Bartonellae* and the exact nature of this regulatory connection must still be examined.

## 4 EVIDENCE SUPPORTING BaGTA‐MEDIATED RECOMBINATION IN VIVO

**Figure 2:**
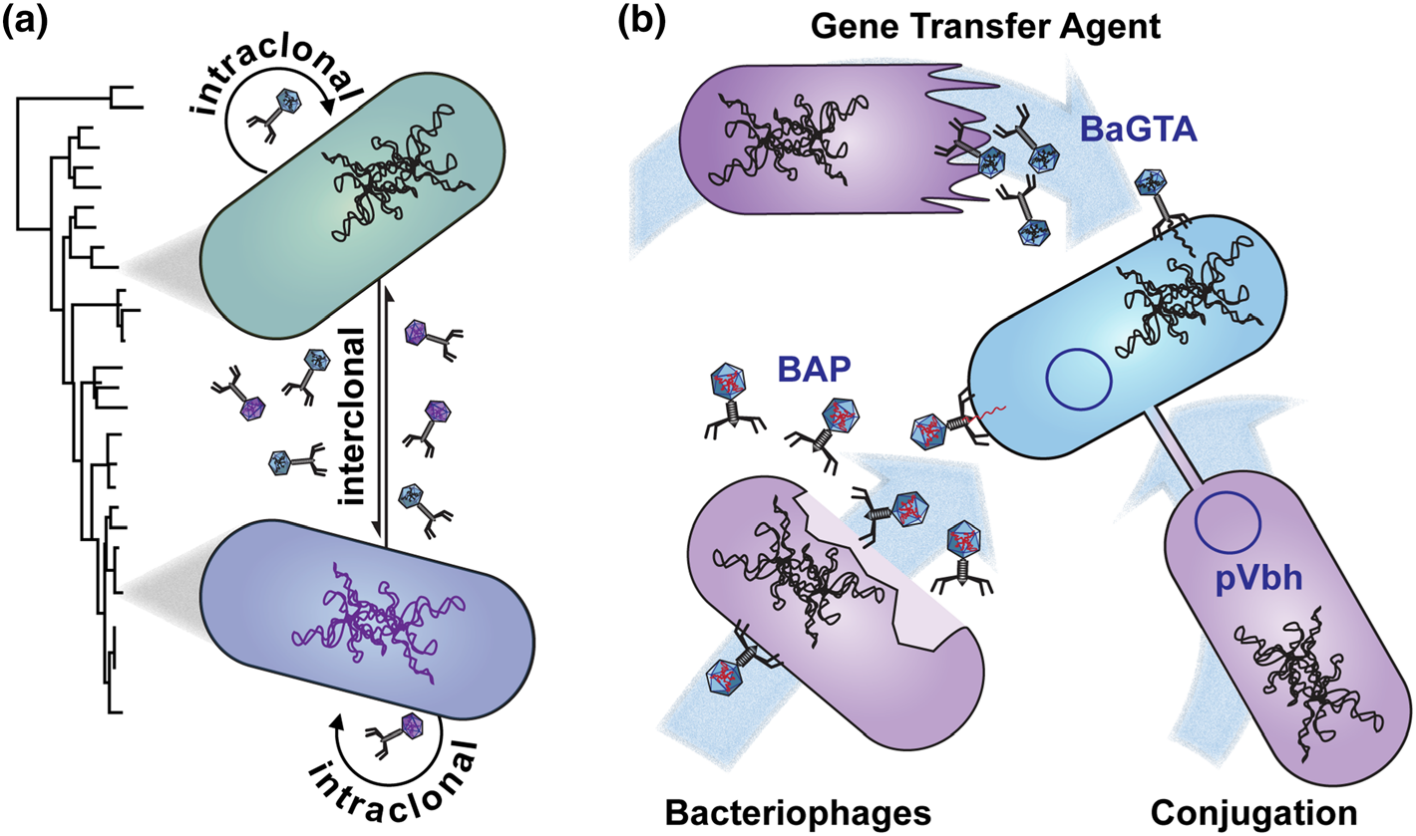
Horizontal gene transfer (HGT) mechanisms *in Bartonella*: function of BaGTA and other mechanisms. (a) Model of the proposed dual mode of action for BaGTA. (i) Evolvability is favoured by interclonal exchanges and may drive the adaptive radiation observed for these zoonotic pathogens, favouring colonisation of new mammalian reservoirs. The midgut of *Bartonella* arthropod vector is a likely niche for such exchanges as it brings together pathogens with different infection history. (ii) Intraclonal recombination counteracts Muller's ratchet and minimises fitness loss resulting from stringent transmission bottlenecks (see main text). (b) Overview of HGT mechanisms at work in the *Bartonellaceae*. Present in all *Bartonella* species, BaGTA is the only conserved source of HGT in that genus. Various temperate bacteriophages have been described in *Bartonella*, including BAP (Alsmark et al., 2004) present in *Bartonella henselae* and others. Importantly, their distribution varies between and within different lineages as well as between different isolates. Finally, an ~30‐kbp conjugative plasmid (pVbh) has been isolated in a subset of the *Bartonella* strain and species (Harms et al., 2017)

Finding the right balance for genome integrity in a clonal population (e.g., Bobay, Traverse, & Ochman, 2015; Bryant, Chewapreecha, & Bentley, 2012; Sheppard, Guttman, & Fitzgerald, 2018) can appear to be a Cornelian dilemma. In fact, although organisms must protect themselves from the irreversible accumulation of deleterious mutations (Muller's ratchet), they can also largely profit from the acquisition of novel beneficial traits, as these may promote adaptive evolution and potentiate colonisation of new ecological niches (Didelot, Walker, Peto, Crook, & Wilson, 2016; Ochman, Lawrence, & Groisman, 2000). BaGTA‐mediated HGT may represent one elegant solution to fulfil both requirements (Figure 2a). Such a dual role represents an asset for *Bartonella*, considering the adverse host(s) environments they are facing and the repeated alternation between transmission bottlenecks and rapid expansion phases within different niches (Chomel et al., 2009; Gutierrez et al., 2015). Remarkably, an in‐vitro evolution experiment performed by repetitive single‐colony passaging of two rodent‐associated *Bartonella* strains revealed low‐level accumulation of single nucleotide variations (in the range of 1 per 1,000 generations; Gutierrez et al., 2018). This experiment reproduces the larger prophage‐driven genomic reorganisations that are commonly seen in natural isolates (Gutierrez, Markus, et al., 2018). However, it poorly reflects the genetic diversity observed at the nucleotide level for rodent‐associated *Bartonella* species sampled from wild animals and their flea hosts (Bai, Kosoy, Lerdthusnee, Peruski, & Richardson, 2009; Gutierrez et al., 2015; Inoue et al., 2009; D. M. Li et al., 2015). It is tempting to speculate that BaGTA accounts for the low mutation rate observed in these intraspecies evolution experiments (clonal stability). Supporting arguments are as follows: (a) Very high frequency of GTA‐mediated intrastrain recombination was described in vitro for *B. henselae* (Québatte et al., 2017), and (b) homologous recombination events in clonal populations are hardly traceable as they do not result in mutations. Although GTAs were already recognised as a valuable means to counteract Muller's ratchet, the actual involvement of BaGTA in this process will require experimental verification.

Besides a possible role in genome homeostasis, BaGTA was already proposed as a likely driver of the observed adaptive radiation that characterizes host‐associated *Bartonellae* (Berglund et al., 2009; Guy et al., 2013; Québatte et al., 2017; Tamarit et al., 2018). In that context, a few points need to be taken into consideration. First, the process of recombination (whether GTA mediated or not) is highly sensitive to the length of precisely base‐paired segments between recombining homologues (Shen & Huang, 1986; Watt, Ingles, Urdea, & Rutter, 1985; and others). As such, recombination efficiency inversely correlates with sequence divergence among homologous sequences or in other words with the genetic distance between organisms. Moreover, rates of homologous recombination vary by several orders of magnitude among bacteria (Vos & Didelot, 2009). Early work based on multilocus sequence typing data estimated a low relative frequency of recombination to mutation (r/m) for *B. henselae* isolates (Vos & Didelot, 2009). However, subsequent genome‐wide studies of recombination rate variation indicated significant recombination frequencies within the natural *B. henselae* population, albeit little novel gene acquisition, which is indicative of a closed pan genome (Guy et al., 2012). This finding naturally brings into question the genetic diversity within the ecological niche(s) occupied by different *Bartonella* species. As it happens, *Bartonella* species face different levels of ecological isolation, which directly reflects on their population dynamics. For instance, diversification by recombination was reported to be high for the rodent pathogen *Bartonella grahamii* in Asia but low in Northern Europe and America (Berglund et al., 2010). This finding suggests that ecological opportunities may drive recombination frequencies between different clonal isolates, at the strain or species level. Importantly, rodents and their associated flees are one of the most important *Bartonella* reservoirs in terms of both incidence and genetic diversity, with more than half of the described species sharing this potentially overlapping niche (Buffet, Kosoy, & Vayssier‐Taussat, 2013; Chomel et al., 2009; Gutierrez et al., 2018). This implies a great potential for HGT‐mediated genetic diversification (gene pool) to these otherwise isolated organisms. The strongest evidence that genetic exchanges actually occur when complex networks of diverse *Bartonella* isolates are found in overlapping habitats came from the identification of numerous recombination events both between and within rodent‐adapted strains coexisting in a small territory of Northern Poland (Paziewska, Harris, Zwolinska, Bajer, & Sinski, 2011). Concordant results were obtained in studies performed on wild *Gerbillus* spp. rodents and their associated fleas in the West Negev region of Israel (Gutierrez, Cohen, et al., 2018; Gutierrez, Morick, Cohen, Hawlena, & Harrus, 2014). In that case, a high rate of multiple coinfections by different *Bartonella* species was observed, and coinfections were directly associated with intraspecies and interspecies recombination (Gutierrez, Cohen, et al., 2018). Whereas these studies primarily focused on recombination events happening in housekeeping genes, other investigations revealed a dramatically increased fixation rate for recombination events in the gene clusters encoding T4SSs and their associated effector proteins (Guy et al., 2012; Paziewska, Sinski, & Harris, 2012; Saenz et al., 2007). This higher fixation rate may result from two nonmutually exclusive phenomena: (a) an increased selection for diversification of these major host adaptability factors that are considered to drive adaptive radiations in the *Bartonellae* (Engel et al., 2011; Harms, Segers, et al., 2017; Saenz et al., 2007) and/or (b) their proximity to BaROR and thereby their likely enhanced HGT via BaGTA (Guy et al., 2013; Québatte et al., 2017). Together, these documented instances of interspecies homologous recombination demonstrate the occurrence of HGT between *Bartonella* isolates sharing overlapping ecological niches. Although they are compatible with the notion of BaGTA‐mediated adaptive evolution, they still do not constitute direct proof of such events.

Prophages and plasmids represent two additional sources of HGT that have been observed in *Bartonella* (Figure 2b). They thus could also contribute to genome diversification by recombination. Prophages have been directly implicated in *Bartonella* genome dynamics, notably by lineage‐specific genomic expansion and reduction or by large genomic rearrangements, even at the species level (Berglund et al., 2009; Berglund et al., 2010; Engel & Dehio, 2009; Gutierrez, Markus, et al., 2018; Guy et al., 2013; Harms, Segers, et al., 2017; Lindroos et al., 2006). Of note, the nature and occurrence of *Bartonella* prophages is very heterogeneous, none of them is conserved throughout the *Bartonellae* or even within sublineages, and strain‐specific variations are observed. Whereas active prophage transduction was monitored by a transposon sequencing approach in *B. henselae*, the transduced material appeared strictly limited to the flanking regions of its chromosomal integration site (Québatte et al., 2017). Further, no evidence for prophage‐mediated generalised transduction in *Bartonella* has been reported to date. From that perspective, and although they are undeniably important components of *Bartonella* genomic plasticity, their contribution to the reported dispersed homologous recombination events may only be marginal. Similarly, the conjugative plasmid pVbh (Harms, Liesch, et al., 2017) is restricted to just a few *Bartonella* strains. Although interspecies pVbh conjugation was demonstrated under laboratory conditions (Harms, Liesch, et al., 2017), its sparse distribution suggests a rather limited contribution to HGT at the genus level. Noteworthy, conjugative systems were once instrumental for *Bartonella* transition towards a mammalian host‐associated lifestyle and their subsequent diversification. Indeed, *Bartonella* T4SSs and associated effector proteins can be traced back to plasmid‐encoded ancestors (Engel et al., 2011; Harms, Segers, et al., 2017; Wagner & Dehio, 2019). Interestingly, the macaque and human‐specific species *Bartonella quintana* contains neither prophages nor plasmids (Alsmark et al., 2004), implying that diversity observed among isolates (Arvand, Raoult, & Feil, 2010; H. Li et al., 2013) could be entirely mediated by BaGTA. Together, these findings suggest that BaGTA‐mediated HGT likely accounts for the majority of recombination events that drive genome diversification and thus adaptive radiation within the genus *Bartonella*.

## 5 CONCLUSIONS AND PERSPECTIVES

Combined with the extensive progresses in *Bartonella* genomics, recent research on BaGTA has provided a wealth of new information on the evolution of these bacteria. Notably, identification of intraspecies and interspecies recombination in *Bartonella* isolates strongly supports the important role of BaGTA‐mediated HGT in their ongoing adaptive evolution. Although compelling, these findings remain largely circumstantial and therefore call for further experimentation. Moreover, many specific aspects of BaGTA activity remain elusive. Importantly, future lines of research should address, among others, the following aspects: (a) Extend the analysis for homologous recombination to whole‐genome sequences, as it was to date largely restricted to genes used for multilocus sequence typing. Targeted analysis on related natural isolates in the light of possible BaGTA‐mediated transfer should further substantiate the determination of recombination rates between strains and species sharing overlapping niches. (b) Determine at which stage of the *Bartonella* life cycle BaGTA is preferentially activated and by which environmental signal(s). In that context, the midgut of the arthropod vectors is a conceptually attractive ecological melting pot, as it brings into close contact different clones—strains or species—that have successfully colonised individual mammalian reservoir hosts. However, a role within the mammalian host should also be considered. (c) Assess the limits of BaGTA‐mediated intraspecies and interspecies HGT and recombination, both in vitro and in vivo. To date, the bacterial host range of BaGTA particles remains elusive; likewise, the nature of the surface receptor required for their adsorption is unknown. Spreading of BaGTA‐contained DNA could be further limited by restriction–modification barriers and by the degree of sequence divergence. Therefore, clarifying these parameters is critical for accurately modelling the impact of BaGTA on gene dissemination in mixed *Bartonella* populations. (d) Investigate the occurrence of the dual activity proposed for BaGTA, that is, counteracting Muller's ratchet versus increasing genetic diversity by using refined in‐vitro and in‐vivo experimental models. Altogether, such studies would pave the way to applying BaGTA‐mediated HGT for experimental evolution experiments in the context of host adaptation.

## CONFLICT OF INTERESTS

The authors declare no conflict of interests.
